# Direct Growth of Carbon Nanotubes on New High-Density 3D Pyramid-Shaped Microelectrode Arrays for Brain-Machine Interfaces

**DOI:** 10.3390/mi7090163

**Published:** 2016-09-08

**Authors:** Bahareh Ghane Motlagh, May Choueib, Alireza Hajhosseini Mesgar, Md. Hasanuzzaman, Mohamad Sawan

**Affiliations:** 1 Polystim Neurotechnologies Laboratory, Department of Electrical Engineering, Polytechnique Montreal, Montreal, QC H3C 3A7, Canada; md.hasanuzzaman@polymtl.ca (M.H.); mohamad.sawan@polymtl.ca (M.S.); 2 Institut Lumière Matière, Université Claude Bernard Lyon 1, CNRS, Univ Lyon, Villeurbanne 69622, France; may.choueib@gmail.com; 3 Microfabrication Laboratory (LMF), Thin Films Group (GCM), Department of Engineering Physics, Polytechnique Montreal, Montreal, QC H3C 3A7, Canada; alirezahm@ieee.org

**Keywords:** brain-machine interface, microelectrode arrays, microfabrication technologies, carbon nanotubes, electrode Impedance

## Abstract

Silicon micromachined, high-density, pyramid-shaped neural microelectrode arrays (MEAs) have been designed and fabricated for intracortical 3D recording and stimulation. The novel architecture of this MEA has made it unique among the currently available micromachined electrode arrays, as it has provided higher density contacts between the electrodes and targeted neural tissue facilitating recording from different depths of the brain. Our novel masking technique enhances uniform tip-exposure for variable-height electrodes and improves process time and cost significantly. The tips of the electrodes have been coated with platinum (Pt). We have reported for the first time a selective direct growth of carbon nanotubes (CNTs) on the tips of 3D MEAs using the Pt coating as a catalyzer. The average impedance of the CNT-coated electrodes at 1 kHz is 14 kΩ. The CNT coating led to a 5-fold decrease of the impedance and a 600-fold increase in charge transfer compared with the Pt electrode.

## 1. Introduction

One of the major goals of the emerging field of neurotechnology is restoration of nervous system disorders. Neuroprosthetic devices that interface with the central nervous system (CNS), called brain-machine interfaces (BMI), enable direct communication with still-functioning parts of the neural pathways and have the potential to restore various lost functions of patients with vision impairment, epilepsy, Parkinson’s disease, or depression [1,2]. Microelectrode arrays (MEAs) are a key element in function restoration as they allow interfacing with neural tissues creating a bioelectronic-neural tissue link. For electrical stimulation and recording, electrodes with a multi-dimensional geometry, high selectivity and density, as well low impedance, are required. Therefore, developing fabrication methods that improve the electrodes electrical and mechanical characteristics are essential.

**Figure 1:**
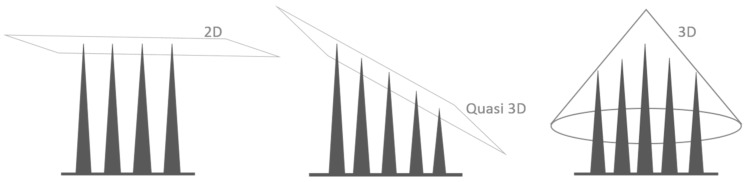
Schematic of the 2D, quasi 3D, and 3D MEAs.

To date, penetrating silicon-based MEAs have been fabricated with two common micromachined architectures: in-plane and out-of-plane. The first architecture—in-plane microelectrodes—was developed at the University of Michigan. In these MEAs, microelectrode contacts are patterned along the shanks. This technology provides a high density of contacts; however, the shanks cause large tissue displacement and may damage a significant number of neurons during insertion [3]. The second MEA architecture—Utah and Utah Slanted electrode arrays—includes sharpened silicon needles electrically isolated from each other [4]. The architecture of these electrodes enables single-unit recording with high-spatial resolution, and excites the neurons by electrical stimulation. The drawback of this MEA architecture is that it is two-dimensional (2D) which only permits recording data from a plane area of the brain. Even the Slanted Utah array is quasi-3D rather than of 3D (Figure 1) [5].

Electrode performance is a compromise between high selectivity that can be obtained by using smaller electrodes and the resulting increase in impedance and lower sensitivity. To address this issue, we should consider the entire combination of main aspects; fabrication method, electrode geometry, and coating materials.

One of the main challenges is finding appropriate coating materials which are biocompatible and improve neural stimulation and recording. Materials with higher charge injection capacity not only enhance smaller electrodes with higher selectivity but also provide a higher stimulation current density and lower stimulation potential.

The in-plane electrodes are sharpened silicon needles that are electrically isolated from each other. The tips of the electrodes are coated with metals and their shank is covered with an insulator.

In order to insulate the body of the electrodes, parylene-C is used due to its biocompatibility, chemical inertness, and high stability in vivo [6]. The high-temperature during annealing causes parylene to evaporate and make it more brittle and predisposed to cracking [7,8]. SiO_2_ deposited by the plasma-enhanced chemical vapor deposition (PECVD) technique is a high-quality, biocompatible, chemically inert, and stable electrical insulator that can resist high temperature and reduce biofouling in a biological environment [9]. Moreover, the perfect adhesion of SiO_2_ to silicon may solve the delamination issue.

The tips of the electrodes need to be good conductors in order to facilitate the charge transfer from the needles to the neural tissues. The active sites of the electrodes are typically coated with Pt or iridium oxide [10,11,12]. Although Pt has excellent electrochemical stability, corrosion resistance, and limited reactivity to the biological environment [13,14], it has a relatively modest charge injection limit (0.1–0.3 mC/cm^2^) [15]. Iridium oxide is a highly conductive oxide with high charge injection capacity. However, it has several shortcomings, including a deterioration of long term stability if used beyond its charge injection limits and a requirement for circuitry to apply an anodic bias during cathodal charge injection [16].

To date, electrode tips are coated by two conventional methods: poking the electrodes through an aluminum foil or using liquid photoresist as a mask. The first method—poking—is a time-consuming process that may damage electrodes during the process, and is operator-dependent [17]. The second method—liquid photoresist mask—also has some limitations. It is a long process with many steps, such as resist coating, vacuum treatment, UV exposure and development, soft and hard bake. More importantly, both methods may not be practical for 3D electrode arrays since they cannot follow a 3D structure and enhance uniform tip-exposure [18].

The remarkable structural, electrical, and mechanical properties of CNTs, such as intrinsically large-surface areas, biocompatibility, extremely high conductance, and high aspect ratios have attracted much interest as a suitable electrode material for neural tissue [19,20].

Up to now, different procedures have been proposed to coat CNTs on neural interfaces, such as electrochemical deposition, electrophoresis, layer-by-layer assembly, and direct growth [21]. Among these techniques, selective direct growth appears to be the most suitable and robust fabrication process. By controlling growth parameters, one can obtain vertical orientation of CNTs with high density, and high reproducibility. The most important advantage of growing CNTs directly on electrodes is the stability and reliability in time compared to CNT films coated using other methods [22]. However, direct growth is a less commonly used process since it requires additional laborious steps. A standard chemical vapor deposition (CVD) process was used by Ansaldo et al. [22] to grow CNTs on a single penetrating microelectrode tip of commercially available platinum-tungsten microwire capable of enduring high temperatures and electroplated with nickel (Ni) as a catalyst. So far, Pt, which is a very common material for bio-medical devices, has rarely been used in CNT synthesis [23,24,25,26]. Standard growth requires depositing common catalyst metals, such as nickel, cobalt, or iron, which are not considered as biocompatible materials. Therefore, purification methods must be employed post-growth to remove the catalyst particles. According to Liu et al. [27] even after post purification, CNTs preserve a significant amount of non-encapsulated or bioavailable metal residue and impurities.

In this study, an in-plane, high-density, pyramid-shaped MEA has been designed for intracortical 3D recording and stimulation. The novel architecture of this MEA enables recording from multiple intracortical depths of the brain and provides more contacts between the electrodes and targeted neural tissue.

A novel masking method was developed to coat the 3D pyramid-shaped MEA. The proposed masking process has several advantages including a single masking step, simpler fabrication process, reducing production time and cost by more than 50%, and a more uniform electrode tip exposure [28]. In order to improve the electrical properties of the MEA, the tips of the electrodes were coated with Pt following a selective direct growth of CNTs. Moreover, the role of CNTs for promoting electrochemical properties of the electrodes is evaluated. The electrochemical properties of the electrodes have been measured using a potentiostat and then compared with fitting results from a circuit model.

## 2. Materials and Methods

### 2.1. Design and Fabrication of Microelectrode Arrays (MEAs)

**Figure 2:**
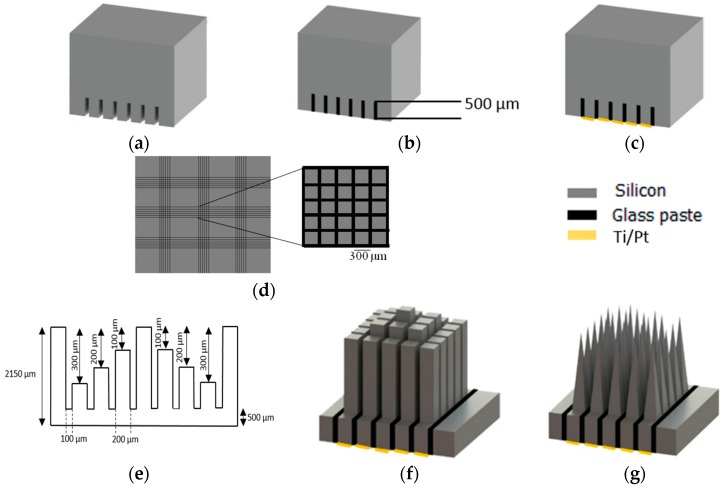
Main steps of silicon-based MEAs micromachining: (**a**) backside dicing; (**b**) backside glassing and polishing; (**c**) backside metallization; (**d**) schematic of top-view of the array backside after dicing and glassing including nine collections of 5 × 5 arrays; (**e**) process flow of dicing variable height electrodes; (**f**) frontside dicing; and (**g**) frontside wet-etching.

The fabrication process of the proposed MEAs consists of two parts: the first one is the design and microfabrication of multi-electrode arrays; the second part is the electrode coating where several techniques and materials are investigated. Figure 2 presents the major steps in the microfabrication of a pyramid-shaped MEA.

The employed substrate representing the base for the probes is a 2150 ± 25 µm thick *p*-type (100) 100 mm-diameter single-side polished silicon wafer with a resistivity of 0.0153–0.0158 Ωcm. To electrically isolate the electrodes from each other, the polished side of the wafer was cut with an Advanced Dicing Technologies (ADT) 7100 dicing saw in two perpendicular directions with the pitch of 300 µm (Figure 2a). A resin-bond blade (B-004-4000J, Dicing Blade Technology, San Jose, CA, USA) was used to make the matrix. The depth of all of the cuts is 500 µm.

To insulate the electrodes from each other, the kerfs have been filled with glass paste (Figure 2b) [29]. To remove the excess glass from the surface of the silicon substrate, the dicing saw and polishing machine were used. The glass was removed with a resin-bond blade (00777-8030-006-QKP, Dicing Blade Technology). Silicon carbide paper and polishing cloth was used to remove the glass residue [17].

To make the electrical connection to each electrode, the backside of each electrode was sputter-coated with a bilayer of metals Ti/Pt (100 nm /400 nm), respectively (Figure 2c, Table 1) [30]. Pt was selected to obtain reliable interconnects, low ohmic contact, and silicide formation. Furthermore, Pt is appropriate for wire-bonding or flip-chip assembly processing. To promote the adhesion of Pt at the silicon surface, a thin layer of Ti was deposited prior to the metal deposition. A lift-off process was used to create a 200 µm × 200 µm contact pad placed between the kerfs filled with glass (Figure 2d).

To achieve electrodes with variable heights, the frontside (non-glassed side) of the substrate was cut with an ADT 7100 dicing saw using two dicing blades: resin-bond blade (J-014-4000-J, Dicing Blade Technology) and a Ni alloy diamond blade (Disco, ZH05-SD2000-M-90). To produce columns with variable heights, silicon was removed from the top of the substrate with the depths of 300, 200, 100, 0, 100, 200, and 300 µm with an index of 300 µm by the resin-bond blade. The substrate was rotated 90° and silicon was removed from the top with the same cutting protocol. The columns with different heights were separated from each other in two perpendicular directions using the Ni alloy diamond blade with the thickness of 100 µm and to a depth of 1650 µm (Figure 2e). The frontside cuts were aligned with the backside ones. This leaves a 7 × 7 matrix of rectangular columns with the height of 1.35, 1.45, 1.55, and 1.65 mm and 100 µm spacing (Figure 2f). The extra row of columns with the height of 1.35 mm was designed as a dummy row to protect electrodes in the arrays and to improve the uniformity of the electrodes during the etching process.

To convert the rectangular columns to sharp tips, a wet etching process composed of 49% hydrofluoric acid and 69% HNO_3_ in a ratio of 1:19, was used [31]. The sample was placed in a 2 cm × 2 cm custom Teflon holder, put into the acid upside down, and then the solution was rotated with a magnetic stirrer at 500 rpm for 5 min [32]. For the following etching step, the sample was placed face down in an etchant solution and N_2_ gas was applied to polish and sharpen the top of the columns until a complete needle shape was achieved (Figure 2g).

### 2.2. Shank Insulation

**Figure 3:**
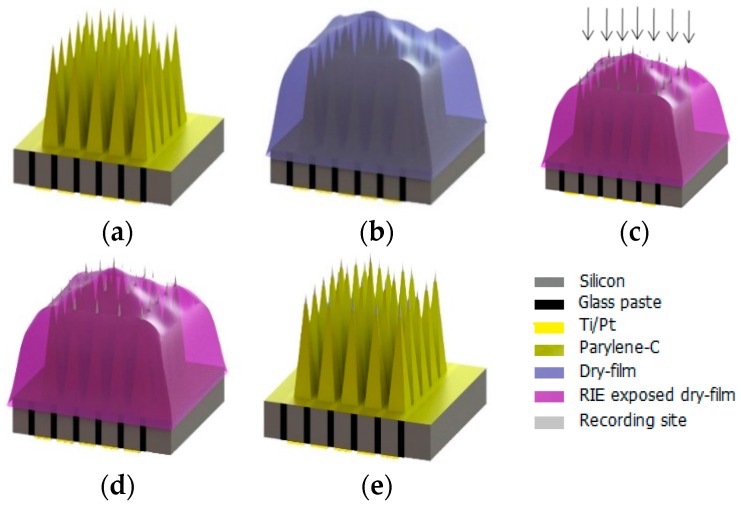
Process flow of Pt electrodes tip-coating: (**a**) Parylene-C deposition; (**b**) cover with dry-film photoresist; (**c**) reactive ion etching; (**d**) tip-metallization; and (**e**) lift-off in acetone.

A 2 µm thick parylene-C film was deposited on the frontside of the electrodes using a CVD process whereas the backside of the electrodes was covered with a tape (Figure 3a) [33]. Parylene-C was deposited using a Specialty Coating Systems (SCS) instrument.

### 2.3. Masking Process

**Figure 4:**
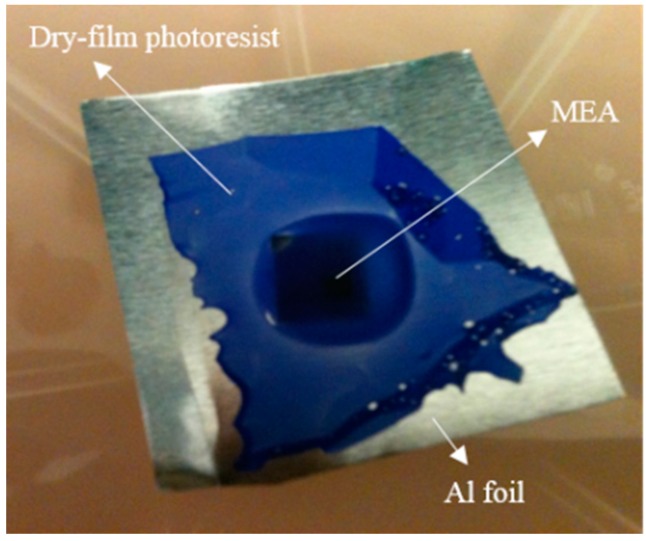
Optical image of masking process using dry-film photoresist as a mask.

After electrode insulation, a layer of dry-film photoresist (FX900, DuPont, Wilmington, DE, USA) with the thickness of 30 µm was used as a mask to cover the array (Figure 3b). The MEA was placed on a thick Al foil sheet. In the next step, the array was covered manually with a dryfilm using tweezers. The dry-film acts as a tape and not only fixes the MEA on the top of the Al foil but also follows the 3D structure of the electrodes, which results in a uniform tip exposure for variable-height electrodes after the etching process (Figure 4).

The dry-film and parylene-C were removed from the tips using isotropic and anisotropic reactive-ion etching processes. In the first step, the dry-film and parylene-C films were anisotropically etched by reactive-ion etching (reactive ion etching (RIE), RF source) from the top of the electrode tips at a power of 200 W and a chamber pressure of 100 mTorr for 50 min. In the next step, both films were etched isotropically with a plasma asher (microwave source) from the side-walls of the tips at a power of 150 W and a chamber pressure of 400 mTorr for 10 min (Figure 3c).

### 2.4. Tip Metallization and Lift-off

The tips of the electrodes were coated with Pt using sputtering (Figure 3d). A thin layer of Ti was deposited prior to the Pt deposition. The deposition of a metal layer at the electrodes’ tip not only decreases its impedance but also facilitates charge transfer from the electrode to the neural tissues. The sputter deposition parameters for Ti and Pt layers are listed in Table 1. The dry-film mask was removed in a lift-off process by ultrasonic cleaning in acetone, isopropanol, and deionized water (Figure 3e).

### 2.5. CNTs Growth

In order to grow CNTs at the tips of the electrodes, another set of MEAs were prepared. A very thin layer of Pt (5–8 nm) was evaporated at the tip of the electrodes prior to CNTs growth. A 2 nm Ti layer was evaporated prior to Pt deposition to improve Pt adhesion and to avoid the Pt diffusion in silicon during CNTs growth. An evaporator was used to deposit very thin layers of Ti and Pt at the tips of the electrodes to better control the deposited thickness. For the electrodes with CNT tips, native SiO_2_ was considered as an insulator instead of parylene-C.

In order to grow CNTs, the catalyst particles formed from the deposited Pt layer must have a nanoscale size (typically <200 nm). It has been previously demonstrated that a Pt film with the thickness of 8.5 nm was unsuccessful for Pt-CNT growth, perhaps because the resulting nanoparticles were too large to support CNT growth [34].

We have carried out CNT growth using PECVD, which is an appropriate growth process for neural interfaces [35]. Spaghetti-like and well-aligned morphologies could be controlled in the CVD process by tuning the thickness of the catalytic metal film. The plasma facilitates the growth of well-aligned, individual, and immobilized nanotubes with uniform diameters [36]. Furthermore, lower growth temperatures relative to CVD are possible since high-energy electrons present in the discharge plasma supply the energy necessary for chemical reactions in the gas [37]. This allows a direct growth of CNTs on soft polymer substrates, for example. Another advantage is that during the growth process, the plasma removes amorphous carbon, which strongly affects the electrical properties of electrodes and may lead to an increase of impedance [25,36].

CNTs were grown in a gas mixture of acetylene (C_2_H_2)_ and ammonia (NH_3_). The MEA is introduced into the furnace and pumped down to a base pressure of 10 mTorr using a mechanical pump. The temperature of the furnace is then ramped up to 700 °C at a rate of 200 °C/min in order to avoid the glass being melted at the backside of electrodes. Before CNT growth, we annealed at 700 °C under 20 Torr of hydrogen. This step allows the formation of catalyst islands from the Pt layer at the tips [36]. After a 10 min annealing, the furnace is pumped down and NH3 is introduced at 3.5 Torr with the temperature held at 700 °C. A DC discharge between the cathode-sample and the anode is initiated and kept at relatively low current (1 mA), while C_2_H_2_ is introduced using a separate mass flow controller. The flow-rate ratio was maintained at 20% (C_2_H_2_/NH_3_, 40/200) which has been found to be the optimum ratio to obtain clean CNTs [36]. The synthesis was carried out for 15 min in a stable discharge.

## 3. Electrochemical Measurements of the MEAs

### 3.1. Electrochemical Impedance Spectroscopy (EIS) and Cyclic Voltammetry (CV)

Electrochemical impedance spectroscopy (EIS) and cyclic voltammetry (CV) were performed using a Biostat VMP-300 system (BioLogic Science Instruments, Grenoble, France). The instrument was operated under computer control with EC-Lab software. A solution of 0.9% phosphate-buffered saline (PBS) was used as the electrolyte in a three-electrode cell consisting of Ag/AgCl as a reference electrode, a large area Pt wire as a counter electrode, and electrodes of the array as working electrodes. The impedance was measured by applying an AC sinusoid waveform with a 10 mV amplitude as the input signal, the DC potential was set to 0 V through the working electrode. The impedance was measured in the frequency range of 40 Hz to 10 kHz. CV was performed with the same instrument and the software. The setup of the electrodes (reference, working, and counting) was the same as the one used in the impedance measurement. The potential on the working electrode was swept between −0.6 V and 0.8 V. The maximum cathodic (Emc) and anodic potentials on the electrode during electrical stimulation should stay within the “water window” (−0.6 V to 0.9 V versus Ag/AgCl in PBS) to prevent electrolysis of water [38,39]. A scan rate of 50 mV/s was used.

### 3.2. Charge Delivery Capacity (Q_CDC_) and Charge Injection Capacity (Q_inj_)

In order to determine the charge delivery capacity (*Q_CDC_*), the electrodes were activated with a repetitive potential cycling at a scan rate of 50 mV/s in the potential range −0.6 to 0.8 V. The *Q_CDC_* was calculated using the following equation [40]:

(1) $$Q_{CDC}=\frac{1}{\nu A}\int_{E_{1}}^{E_{2}}\left|i\right|dE\;(\mathrm{mC}/{\mathrm{cm}}^{2})$$

where ν is the corresponding scan rate (mV/s), *E* is the electrode potential (V), *E*_1_ and *E*_2_ are the anodic and cathodic potential limits (V), *A* is the surface area of the electrode tip (cm^2^), and *i* is the measured current (A).

The surface area of the electrode’s active sites is difficult to measure due to surface roughness and porosity. Therefore, the geometrical surface area of the electrode tip was used to determine their *Q_CDC_*. The lateral surface area (LSA) of the active site with a tip-exposure of 50 µm was calculated by the following equation:

(2) $$LSA=\pi r\;\sqrt{h^{2}+r^{2}}$$

where *r* and *h* are the radius and height of the cone, respectively.

To measure the charge injection capacity (*Q_inj_*) of the microelectrodes, we interfaced the MEAs with a novel microstimulator designed in our laboratory [41,42]. The MEA was immersed in 0.9% PBS solution, then charge-balanced and biphasic constant current was applied to different pairs of microelectrodes. Stimulation currents with the range of 10 to 110 µA were applied for pulse widths of 0.7, 0.8, and 1 ms. The stimulation frequencies were set to 500 Hz, 500 Hz, and 250 Hz, respectively. The resulting waveforms were measured across each pair of microelectrodes using a digital oscilloscope. The area under each pulse was estimated for determining the total charge injected during any anodic or cathodic pulse and divided by the electrode surface tip area.

### 3.3. Equivalent Circuit Model

**Figure 5:**
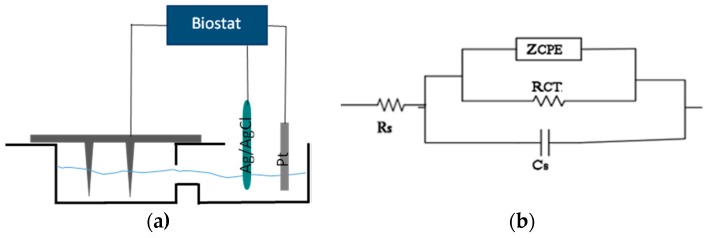
Electrochemical measurement setup: (**a**) MEAs measurement setup; (**b**) Equivalent circuit of the electrode-electrolyte interface.

The equivalent circuit model includes a constant phase element impedance (ZCPE) that represents the electrode’s capacitive impedance shunted by a charge transfer resistance RCT, both in series with the solution resistance *Rs*. *Cs* is all the shunt capacitance to the ground comprises the capacitance from the metal of the electrode to the solution through the insulation (Figure 5) [43,44]. When the current is applied to the electrode-electrolyte interface, a double-layer of charges simulates a charged capacitor. The double-layer capacitance occurs from the movement of charges and ions at the interface. Layers of charges are formed by two mechanisms; Faradic and non-Faradic transfer. Faradic reactions can be reversible or non-reversible and are undesirable for biological purposes since the tissue is altered during the oxidation and reduction processes resulting in pH changes that can damage the tissue and the electrodes. Non-Faradic charge transfer is a reversible process and is defined by the motion of charges at the interface to form a charge separation region which is the origin of the double layer capacitance. The latter can be modeled as a parallel-plate capacitor whose capacitance is given by:

*C_s_*=εε_0_*S*/*d* (3)

where ε is the dielectric constant of the medium and ε_0_ is the dielectric permittivity of vacuum, *S* is the surface area, and *d* is the distance between charge layers.

Non-faradic capacitance is empirically represented by a constant phase element (CPE). The CPE impedance is given by:

(4) $$Z_{CPE}=\frac{1}{A{\left(i\omega \right)}^{\alpha }}\;,\;0\leq \alpha \leq 1$$

where ω is the angular frequency (ω = 2π*f*) and *A* is the measure of the magnitude of *Z_CPE_*. The CPE describes an ideal capacitor for α = 1 and ideal resistor for α = 0. The impedance is purely imaginary for a capacitor and real for a resistor. For a combination of a capacitor and a resistor, a large phase angle indicates that the impedance is mainly capacitive and for the small phase angle values it is resistive [45].

## 4. Results

### 4.1. Characterization of the 3D MEAs

**Figure 6:**
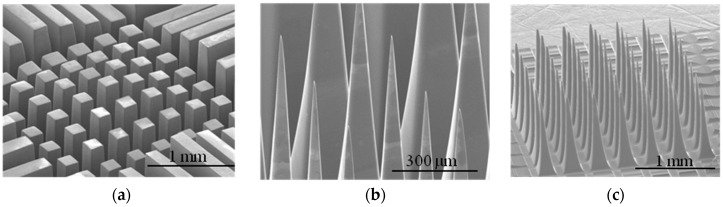
SEM images of electrodes frontside: (**a**) 3D pyramid-shaped electrode array with three different heights. The outer row of electrodes is for etching process uniformity; (**b**) polishing and sharpening the tips of electrodes by applying N_2_ gas from the bottom of the solution; and (**c**) one 3D 7 × 7 MEA. The width of the electrodes was 200 μm at the base and about 2 μm at the tip with 100 μm spacing after etching step.

SEM images of a 7 × 7 matrix of rectangular columns with different heights were acquired after the backside dicing and glassing processes (Figure 6a) [28]. The rectangular columns of the electrodes were converted to sharp needle-shaped tips (Figure 6b,c). Etching duration causes significant change in the geometry of the electrodes.

### 4.2. Masking Process

**Figure 7:**
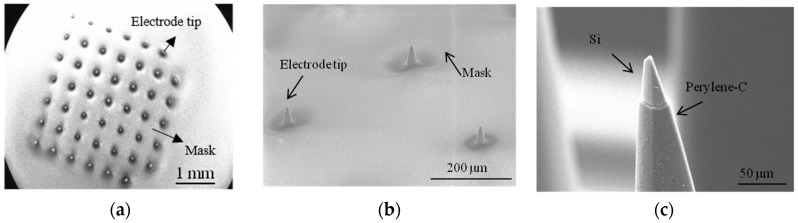
SEM images of masking process: (**a**) electrode array covered with dry-film photoresist after RIE process. Dry-film photoresist follows the 3D structure of the electrodes; (**b**) uniform tip-exposure of variable-height electrodes after RIE and plasma asher etching process; and (**c**) electrode-tip after removing the mask.

Figure 7 shows SEM images of the dry-film deposition following the etching process of the tips and removing the mask ultrasonically.

As indicated above, the novel proposed technique includes a single masking process and could significantly reduce the number of fabrication steps. In this technique, instead of two photolithography steps, which are time-consuming (total processing time: 24 h) there is only one etching step with a total processing time of 6 h. Additionally, none of the conventional methods may be ideal to enhance uniform tip-exposure for 3D structure of the MEA (Table 2) [32]. The non-uniformity (%) in tip-exposure in a 5 × 5 array was 1% ± 0.3%. It should be noted that frontside dicing to cut the electrodes with different heights adds no more than 10 min to the fabrication procedure. This step is performed automatically by programming the dicing saw. Therefore, the effective time-saving of our fabrication procedure is about 18 h.

### 4.3. Tip-Metal Deposition

**Figure 8:**
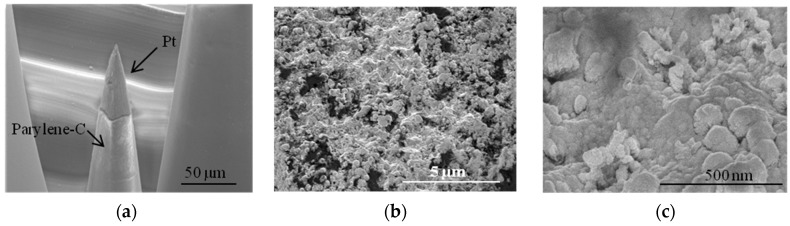
SEM images of microelectrodes after tip-metallization with Pt (**a**–**c**) at different magnification. Pt has granular morphology.

SEM images of a microelectrode tip after coating with Pt is shown in Figure 8 at different magnifications. The thickness of the metals at the tip of the electrodes may differ from the wafer due to the nonplanar shape of the electrodes.

### 4.4. Tip-CNT Growth

**Figure 9:**
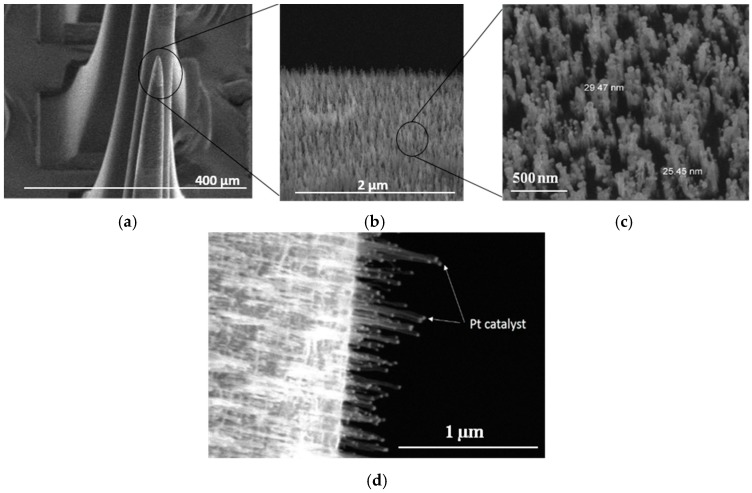
SEM images of microelectrodes after tip-coating with CNTs: (**a**–**c**) at different magnifications. The geometry of CNTs increased the accessible surface area; and (**d**) Pt particles at the end of CNTs.

**Figure 10:**
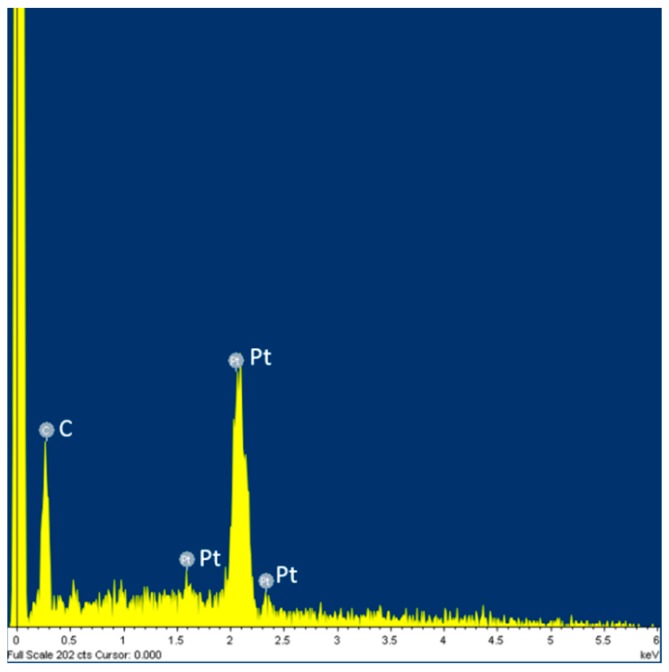
EDX spectroscopy of CNT electrodes with Pt particles at the end of CNTs.

Figure 9 shows SEM images of a MEA after CNT growth. A zoomed-in image of the electrode tip shows forest-like vertically-aligned CNTs characterized by a typical length in the order of 600 nm and a diameter between 25 nm and 30 nm. We can distinguish the presence of Pt particles at the end of CNTs (Figure 9c). It is noteworthy that the length of the CNTs can be varied by tuning the PECVD growth conditions such as temperature, pressure, time of growth, etc. [36]. Energy dispersive X-ray (EDX) spectroscopy confirms the presence of Pt and C at the tip of the electrodes (Figure 10).

### 4.5. Electrochemical Properties of the MEAs

**Figure 11:**
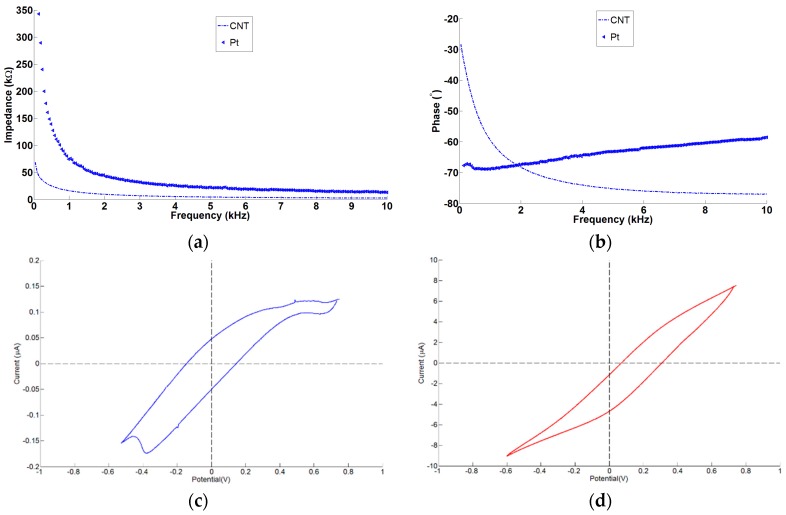
Impedance spectroscopy and cyclic voltammetry of Pt- and CNT-coated electrodes: (**a**) magnitude of impedance as a function of frequency; (**b**) phase of the impedance as a function of frequency; (**c**) CV curves for Pt-coated electrodes; and (**d**) CNT-coated electrodes under similar conditions.

The average impedance at the biologically relevant frequency of 1 kHz of bare silicon, Pt- and CNT-coated electrodes was 850 ± 10 kΩ, 70 ± 0.2 kΩ, and 14 ± 0.2 kΩ, respectively (Figure 11a,b). On average the CNT coating lowered the impedance of Pt-coated electrodes by a factor of five at 1 kHz, and increased the charge delivery capacity by a factor of 600. The CV graph of Pt and CNT has shown that the CNT has a significant larger area than Pt electrodes indicating higher *Q_CDC_* of CNT electrodes (Figure 11c,d).

**Figure 12:**
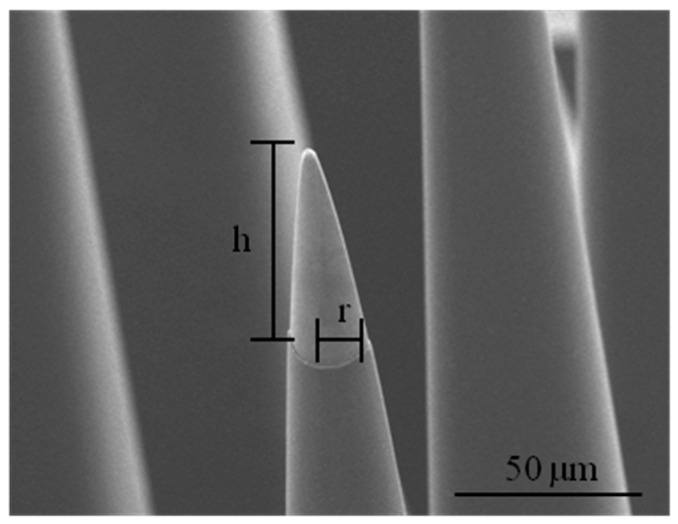
Electrode tip dimensions; the height (*h*) and radius (*r*) of the tip.

The LSA of the electrode tips was calculated to be about 1.6 × 10^−5^ cm^2^ for a tip exposure (*h*) of 50 µm (Figure 12).

The average cathodic charge delivery capacity of Pt- and CNT-coated MEAs were 21 ± 10 mC/cm^2^ and 13,020 ± 6510 mC/cm^2^, respectively. Therefore, there is a significant improvement in *Q_CDC_* when the electrode tips were coated with CNTs.

The calculated average of *Q_inj_* for the Pt- and CNT-coated microelectrodes is 0.3 and 10 mC/cm^2^, respectively, for a stimulation current of 92 µA and pulse-width of 0.8 ms.

### 4.6. Equivalent Electrode-Electrolyte Interface Circuit Model

**Figure 13:**
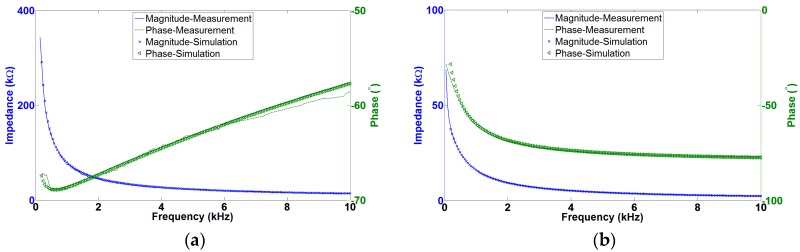
Simulation and experimental results of equivalent electrode-electrolyte interface: (**a**) Pt-tips; and (**b**) CNT-tips.

Increasing the roughness and surface area of the electrodes decreases α due to the increase of atomic scale heterogeneity on a rougher surface. The Faradic process involves transfer of electrons across the electrode-electrolyte interface and was modeled as an electrical resistor (*R_CT_*). The resistance is inversely proportional to the surface area (*R* = ρ*l*/*S*), therefore larger S would result in smaller resistance. Micrographs in Figure 8 and Figure 9 confirm differences in surface roughness and clearly indicate that CNTs have a significantly larger surface area compared with Pt. CNTs donate to an increase in capacitive charge transfer mechanism. Increasing the surface area by CNTs is not the same as roughening the surface area with Faradaic materials, such as Pt. The proposed technology claims to have Pt nanoparticles exposed on the tip of the CNTs. Fitting parameters are given in Table 3, while simulated plots using these parameters are shown in Figure 13.

An equivalent circuit model has been used to describe the electrode-electrolyte interface impedance using electrochemical impedance spectroscopy. The electrolyte resistance for all the coatings was 2.2 kΩ. As it has been shown in Table 3, Pt-coated MEAs are smoother (α) than CNT-coated while the capacitance (A) is lower. The resistance (*R_CT_*) of CNTs is significantly smaller than that of Pt electrodes. The resistance and capacitance of these coatings could be related to the effective surface area. The double layer capacitance around the interface is proportional to the active site surface area (Equation (3)). The large surface area would result in a higher capacitance than in the case of CNTs. The resistance is inversely proportional to the surface area so a larger surface area would result in a smaller resistance. A good match between measured results and the curves generated using the equivalent circuit model indicates that the appropriate model has been chosen. A comparison of electrochemical properties of current available microelectrodes and this work are listed in Table 4.

## 5. Discussions

Comparing this microelectrode with currently available intracortical penetrating MEAs, the presented MEA offers a 3D high electrode-density (25 electrodes/1.96 mm^2^), with lower impedance and higher charge injection capacity, which are desired for neural stimulation. The importance of such pyramid-shaped MEAs has not been quantified yet but may have some advantages during electrode insertion into the tissue e.g., kill less neurons during insertion. A new fabrication technology has several advantages including different possible geometries (3D structure with variable-height electrodes) and ease of fabrication. The usage of the novel masking method not only resulted in uniform tip-exposure for variable-height electrodes but also reduced the lead time and cost of fabrication significantly. It would take 6 h compared to 24 h by the conventional masking method [32,48].

EIS measurements have been done in vitro to characterize the quality of the microelectrodes coated with Pt and CNTs. Pt electrodes have lower impedance compared with currently available microelectrodes. The measured impedance of Pt-coated microelectrodes was 70 kΩ at 1 kHz.

CNT-electrodes exhibited better electrochemical properties compared to Pt. The impedance of CNT-electrodes at 1 kHz was five times smaller than Pt following 600 times higher *Q_CDC_*. More importantly, CNT *Q_inj_* is 33 times higher than that of Pt. Iridium oxide electrodes were not considered because of harmful pH changes during electrical stimulation [40].

Ansaldo et al. have studied electrical properties of three different coatings on sharp Pt/tungsten wire microelectrodes, including CVD growth of CNTs, polypyrrole-CNT (PPy-CNT) and gold-CNT (Au-CNT) composites by the electrochemical co-deposition method [20]. The authors have shown that the PPy-CNT improved the *Q_CDC_* and lowered impedance compared with CVD-CNT electrodes; however, PPy-CNT’s electrical properties degrade with time (Table 4).

Abidian et al. deposited PEDOT nanotubes on planar neural electrodes and have measured the charge capacity density (*Q_CCD_*) and impedance which were about 110 mC/cm^2^ and 10 kΩ at 1 kHz, respectively [49]. The authors have later shown that the combination of PEDOT nanotubes with PEDOT and hydrogel improved the *Q_CCD_* to 220 mC/cm^2^ and lowered impedance to about 2.5 kΩ at 1 kHz. The impedance of PEDOT + PEDOT nanotubes + hydrogel is less than CVD-CNT; however, the coating process of PEDOT with different combinations is more complicated compared with CVD-CNT.

The direct growth of CNTs at the tips of presented microelectrodes could increase the *Q_CDC_* five times more than PPY-CNT electrodes. The *Q_CDC_* of this MEA is even higher than electrodes coated with polymers and hydrogels which involve hydrophilicity.

Another parameter which is important to compare the MEAs materials for neural devices is *Q_inj_*. The *Q_CDC_* of materials can be increased by increasing the surface area but it may not increase the charge injection capacity [50,51]. As mentioned above the *Q_CDC_* of direct growth of CNT-electrodes is 13,020.1 ± 6510.2 mC/cm^2^ while the *Q_inj_* is only 10 mC/cm^2^. The *Q_inj_* of presented MEA is higher than PPY-CNT electrodes.

In order to improve biocompatibility of the MEAs, the surface of CNT-coated electrodes were coated with polyethylene glycol (PEG) and poly-d-lysine (PDL) peptide. PEG-coated MEAs were immersed in solution of 0.1 mg/mL PDL and 0.1 M PBS. The solutions, including electrodes, were magnetically stirred at 100 rpm for 24 h. In vitro cell culture tests were performed to evaluate the growth of neuroblast cells on the CNT-coated electrodes. MEAs were cultured with mouse neuroblast cells for four days and monitored after 6, 12, 24, 48 h, and 4 days. Cells grew and proliferated normally in the presence of Pt and CNT-coated electrodes. CNTs remained mechanically stable and attached to the electrode tips after PDL deposition and cell culture process [52].

These materials not only improved the biological compatibility of the electrodes but can also be absorbed to the CNTs surface through noncovalent interactions. The CNT has an inherently large surface area but most of its surface area is inaccessible in electrolyte aqueous solution and cannot contribute to charge injection. Various surface modification techniques exist to enhance the hydrophilicity of the CNTs electrodes. One of the techniques to modify CNTs is coating electrodes with peptides. The peptide binds strongly to the nanotube side wall via van der Waals and hydrophobic interactions, while the PEG chain extends into the water. As a result, the CNT-coated microelectrodes turned more hydrophilic. Another hydrophobic to hydrophilic transition happens during incubating electrodes with cell culture medium or a plasma treatment. Wang et al. has shown that functionalized hydrophilic CNT microelectrodes could increase effective surface area about 300 times and enhance high charge injection during stimulation [15].

## 6. Conclusions

A novel, high-density, penetrating, pyramid-shaped microelectrode array for recording and stimulation neurons was designed and implemented. Due to its geometry, the proposed 3D MEA provides higher contact density than available electrode arrays and may allow stimulation at different depths in the cortex. The fabrication technology, described in this work, is simpler and faster than currently available techniques. Additionally, the novel masking technology provides a uniform tip exposure for 3D structures. In addition, selective direct growth of CNTs on the 3D MEA tips using Pt as a catalyst material has been done. On average CNT coating lowered the impedance of Pt-coated electrodes by a factor of 5 at 1 kHz and increased charge transfer by a factor of 600. In vitro cell culture test confirmed mechanical stability of CNTs at the tips of the electrodes. However, further investigations are needed to confirm in vivo stability of CNT-electrodes. The next step will be to perform surface modification to enhance the hydrophilicity of the CNT in order to improve electrochemical properties of the electrodes.

## Figures and Tables

**Table 1:** Sputter deposition parameters for the electrode tip-metallization.

Metal	Ambient	Chamber Pressure (mTorr)	Power (W)	Time (min)	Thickness (nm)
Ti	Ar	10	90	11	100 ± 5
Pt	Ar	10	90	16	400 ± 10

**Table 2:** Comparison of conventional and proposed masking processes.

Conventional Method [32]	This Work
First Masking Process: - Photoresist coating - Vacuum degassing 30 min - Pre-baked - UV exposure - Developing - Hard-bake 7 h - Tip-Metallization - Lift-off	Single masking process: - Parylene-C deposition - Dry-film deposition - Soft-bake - Plasma etch - Tip-Metallization - Lift-off
Second Masking Process: - Parylene-C deposition - Photoresist coating - Vacuum 30 min - Hard-bake 10 h - Plasma etch - Lift-off	

**Table 3:** Fitting results from the EIS model.

Coating	α	*A* (F)	*R_CT_* (kΩ)	*C_s_* (F)
Pt	0.85	2.30 × 10^−8^	1900	9 × 10^−11^
CNTs	0.77	7.20 × 10^−6^	24	8.60 × 10^−9^

**Table 4:** Comparison of penetrating neural MEAs.

MEAs	Geometry	Characteristics				
		Tip-Coating	*Z* (kΩ) at 1 kHz	*Q_inj_* (mC/cm^2^)	*Q_CDC_* (mC/cm^2^)	*Density* (Electrodes/1.96 mm^2^)
[46,47]	2D Quasi-3D	Pt, SIROF ^1^, CNTs ^2^	Pt: 125 SIROF: 6 CNTs: 49.7 Electrode tip surface area: 2 × 10^−5^ cm^2^	Pt: 0.3 SIROF: 2 CNTs ^3^	Pt: 4.4 ± 3.1 SIRO: 34.3 ± 21.7 CNTs: 1217.359 nC	16
[22]	NA	Pt/W ^4^ microwire	CVD-CNT: 15 PPy-CNT ^5^: 7 Au-CNT: 50 Electrode tip surface area: 6.5 × 10^−5^ cm^2^	CVD-CNT: 4 PPy-CNT: 7 Au-CNT: 0.8	CVD-CNT: 1743.4 ± 161.7 PPy-CNT: 2920.3 ± 191.4	Single probe
This work	3D	Pt, CNTs ^6^	Pt: 70 CVD-CNTs: 14 Electrode tip surface area: 1.6 × 10^−5^ cm^2^	Pt: 0.3 CNTs: 10	Pt: 21.7 ± 10.2 CNTs: 13020 ± 6510.2	25

^1^ SIROF: sputtered iridium oxide film; ^2^ CNTs: electrochemical deposition; ^3^ CNTs: cathodal charge storage capacity (QCCSC) was reported instead of charge injection capacity; ^4^ W: tungsten; ^5^ PPy-CNT: polypyrrole-CNT; ^6^ CNTs: direct growth.
